# Multi-class: spectral-spatial temporal pyramid network and multi-class classifier-based cardiovascular disease classification

**DOI:** 10.3389/fphys.2025.1650134

**Published:** 2025-10-24

**Authors:** S. K. Reehana, S. P. Siddique Ibrahim

**Affiliations:** School of Computer Science and Engineering, VIT-AP University, Amaravati, India

**Keywords:** cardiovascular disease, spectral spatial temporal convolutional pyramid network, multi-modality, multi-class classifier, weight correction module

## Abstract

Cardiovascular Disease (CVD) epitomizes class of disorders that disturb the vessels of heart and blood, encircling circumstances such as heart failure, coronary artery disease, and strokes, and leftovers a foremost global cause of morbidity and mortality. The early diagnosis of CVD is decisive as it consents for opportune involvement and organization, plummeting the risk of complications, improving treatment outcomes, and preventing further progression of the disease, ultimately contributing to better patient outcomes and overall cardiovascular health. Furthermore, early detection and diagnosis of CVD benefit significantly from the utilization of electrocardiograms (ECGs) and phonocardiograms (PCGs). The application of DL algorithms for identifying CVDs using PCG and ECG data has gained substantial attention, although a predominant number of existing approaches hinge on data sourced from a single modality. Henceforth, the development of proficient multi-modal Machine Learning (ML) techniques is crucial for effective prediction and detection of CVD. In this paper, we have proposed multi-modality-based CVD diagnosis framework named as multi-class model. In order to classify cardiovascular diseases into several categories using structured clinical data, this study introduces MCC-CVD, a new multi-component deep learning model. A real-world dataset of 920 patient records was used to assess the model. This dataset contains 13 clinical parameters, such as age, cholesterol level, resting blood pressure, fasting blood sugar, and other risk markers. The model used a two-stage weight correction technique and a tri-pattern attention mechanism (TPAM) to achieve robust performance, which allowed for more subtle feature weighting and better interpretability. Here, we utilized both quality enhanced ECG and PCG data through performing multiple processes including noise reduction and normalization. Besides, to evade misclassification data enhancement in terms of false peak elimination is performed based on adaptive thresholding features. After that, we fed the processed data into a multi-class architecture made up of three modules following. For extracting appropriate features, we designed Spectral Spatial Temporal Pyramid Network (SST-PNet) module. Additionally, Weight Correction Module with Attention Mechanism (WCM-AM) employs for weight maximum approach with three-pattern attention mechanism. Finally, novel Multi-class EnDe-CNN classifier is introduced to classify various CVD in multiple classes. A stratified 10-fold cross-validation method was used to carry out extensive studies. Outperforming baseline classifiers like SVM, Random Forest, and Logistic Regression, the suggested MCC-CVD model attained an average accuracy of 92.4%, F1-score of 0.87, precision of 0.89, and recall of 0.85. With an area under the curve (AUC) of 0.94, the model clearly has good discriminative potential across various subtypes of CVD. Furthermore, sensitivity analysis showed consistent performance even when changing parameters or data, and statistical testing validated the model’s superiority with p-values less than 0.05.

## 1 Introduction

Cardiovascular disease (CVD) encompasses a range of conditions affecting the blood vessels of the heart and can lead to serious outcomes like stroke and heart failure. It is a major global health concern due to its high rates of morbidity and mortality (Vinay et al., 2024) (Aggarwal et al., 2021). CVD is often caused by a combination of lifestyle factors, such as poor diet, lack of exercise, and smoking, as well as by genetic predispositions. These conditions typically result in blood vessel narrowing or blocking, which may disrupt the essential flow of oxygen and nutrients to the heart. Timely intervention through lifestyle changes, medication, or surgery is vital for managing cardiovascular health and preventing severe outcomes (Khan et al., 2024) (Chen et al., 2024). Early detection of CVD is crucial for several reasons. First, heart attacks and strokes are leading causes of death worldwide. Identifying these issues early significantly enhances the likelihood of successful treatment and recovery (Khan and Algarni, 2020). Second, many cardiovascular problems develop silently, without noticeable symptoms, until they become severe. Regular screenings and early detection allow for the identification of risk factors and abnormalities before they lead to serious complications. This proactive approach enables the implementation of preventive measures such as changes in diet, increased physical activity, and medication, to manage and reduce CVD risks (Bakkouri and Afdel, 2023) (Kambhampati and Ramkumar, 2021). Additionally, early detection can reduce healthcare costs. Advanced CVD treatments can be resource-intensive and expensive. Identifying and addressing cardiovascular problems early helps allocate healthcare resources more efficiently, leading to better patient outcomes and a more sustainable healthcare system (Bakkouri et al., 2022) (Bakkouri and Afdel, 2020). ECG and PCG are valuable tools for early CVD detection. ECG measures the electrical activity of the heart, captures the rhythm, and identifies irregularities such as arrhythmias (Bakkouri and Afdel, 2019) (Narayana et al., 2023a). The PCG records the heart sounds, providing insights into mechanical activities such as valve movements. Combining ECG and PCG data enhances CVD detection by providing a more comprehensive view of cardiac health. Machine learning (ML) and deep learning (DL) algorithms are increasingly used to analyze ECG and PCG data (Thomas and Kurian, 2022) (Narayana et al., 2023b). These technologies improve the accuracy and timeliness of CVD detection by identifying patterns and abnormalities that may not be evident to human observers. AI-based systems, particularly DL models, excel at processing complex data and can continuously improve their performance as they learn from large datasets (Li P. et al., 2021). This integration of AI with ECG and PCG data offers a promising approach for preventive care and personalized treatment. Despite their potential, there are limitations to using AI for CVD detection, including the need for large, diverse datasets for effective model training, potential biases, and difficulties in interpreting complex or subtle abnormalities (Khan et al., 2022), (Jyothi and Pradeepini, 2024). Additionally, DL models often operate as “black boxes,” making it challenging to explain their decisions to clinicians. Addressing these challenges and adapting to the dynamic nature of cardiovascular conditions is essential for developing robust and reliable diagnostic tools. In summary, early detection of CVD through advanced technologies like ECG, PCG, and AI can significantly improve patient outcomes, reduce healthcare costs, and enhance overall cardiovascular health. The development of new models that integrate these technologies is crucial for refining CVD diagnosis and treatment (Do et al., 2023; Khan et al., 2021; Chakir et al., 2020).

### 1.1 Research contribution

To ensure accurate CVD diagnosis and enhance model performance, the following novel contributions are explicitly introduced in this work.

#### 1.1.1 Enhanced data processing and feature extraction

The framework introduces a robust preprocessing pipeline for ECG and PCG data that includes quality enhancement, noise reduction, and normalization. By performing data enhancement with adaptive thresholding to eliminate false peaks, the model ensures higher accuracy and reliability in feature extraction. The Spectral Spatial Temporal Pyramid Network (SST-PNet) module then leverages such refined data to capture complex spectral, spatial, and temporal features, improving the model’s ability to detect and differentiate various CVD conditions.

#### 1.1.2 Advanced weight correction and attention mechanism

The Weight Correction Module with Attention Mechanism (WCM-AM) represents a novel approach by integrating a three-pattern attention mechanism with weight correction strategies. This module enhances the model’s ability to focus on critical features and corrects weight imbalances, leading to more precise and context-aware classification. This mechanism improves the handling of diverse data patterns and reduces misclassification rates by addressing the nuances of different CVD types. There is a synergistic and complimentary benefit to integrating spectral spatial and temporal data in the context of cardiovascular disease classification. The correlation between cholesterol levels, blood pressure, and other biomarkers is just one example of how spectral–spatial features capture the distributional patterns and interrelationships among clinical variables at a single point in time. This helps the model learn how these attributes co-exist and interact across different patient subgroups. When it comes to cardiovascular disease, temporal aspects are crucial for recognizing early warning signals, diagnosing deteriorating situations, and discriminating between acute and chronic forms. These features encode the progression and evolution of patient health indicators throughout time. The MCC-CVD framework uses combined modeling of both domains to provide more accurate and discriminative depictions of patient’s health conditions by utilizing both static diagnostic indicators and dynamic patterns of disease progression. When it comes to cardiovascular disease (CVD) risk stratification, where both the current clinical profile and past trends help with correct diagnosis and prognosis, this multi-domain fusion is very useful.

#### 1.1.3 Innovative multi-class EnDe-CNN classifier

The introduction of the multi-class EnDe-CNN classifier holds significance as it combines advanced encoding and decoding techniques within a convolutional neural network framework to classify various CVD types across multiple classes. This classifier not only enhances the model’s diagnostic capabilities but also facilitates a more granular understanding of cardiovascular conditions, enabling more targeted and effective treatment strategies. Classical machine learning methods like Support Vector Machine (SVM), Random Forests (RF), and Logistic Regression (LR) are used in the majority of extant work in this domain. These methods usually see CVD prediction as a problem of binary classification, like presence vs. absence of disease. In multiclass classification problems, where different types of cardiovascular diseases are to be distinguished, these approaches frequently fail, despite their moderate performance. On top of that, a lot of models are not ideal for real-time deployment, do not have interpretability, or are overfitted to tiny datasets. Parameter optimization, statistical validation, and scalability to real-world settings have frequently been overlooked in the current applications of deep learning models.

Using structured clinical data, this project aims to build a deep learning model for multiclass cardiovascular illness categorization that is robust, interpretable, and scalable. Multi-Component Classifier for Cardiovascular Disease (MCC-CVD) is the name of the suggested model that improves feature importance learning and classification accuracy by combining a two-stage Weight Correction Strategy with a new Tri-Pattern Attention Mechanism (TPAM). With the use of dynamic attention and statistical rigor, the suggested architecture outperforms previous models that depend on static feature importance or shallow categorization, resulting in improved dependability and wider applicability.

The absence of multiclass CVD prediction models, inadequate statistical evaluation, model interpretability, and cross-platform scalability are some of the important gaps that this study fills. This gap is addressed by the MCC-CVD model, which can (1) process complex, high-dimensional clinical data using deep attention-based learning; (2) deliver strong results supported by cross-validation, confidence intervals, and significance testing; and (3) show that it is feasible to deploy using sensitivity analysis and a design that is efficient with resources.

The main contributions of this study are as follows:

• Development of a novel deep learning architecture for multiclass CVD classification using clinical data.

• Introduction of the Tri-Pattern Attention Mechanism (TPAM) and Weight Correction Strategy to improve feature weighting and interpretability.

• Comprehensive statistical evaluation including 10-fold cross validation, confidence intervals, and paired significance testing.

• Sensitivity and robustness analysis demonstrating model stability under parameter and data shifts.

• Discussion of deployment strategies including model compression and potential integration with mobile apps, cloud APIs, and hospital information systems.

### 1.2 Paper organization

The subsequent tasks are organized as follows: Section 2 elucidates the associated endeavors, providing an in-depth overview of the existing CVD diagnosis model and delineating its research gaps. Section 3 underscores the research methodology, accompanied by pertinent theoretical, diagrammatical, and mathematical elucidations. In Section 4, the experimental results are disclosed, encompassing dataset particulars and evaluation outcomes juxtaposed with the latest iterations of CVD diagnosis models. Section 5 concludes the proposed research.

## 2 Literature survey

CVD remains the leading cause of death globally, underscoring the urgent need for accurate and early diagnosis. Traditional diagnostic methods often rely on single-modality data, which may fail to capture the complex interplay of electrical and mechanical cardiac functions. The integration of ECG and PCG signals offers a more comprehensive view of cardiac health, enabling enhanced detection of subtle abnormalities. With the rise of deep learning, there is a growing shift toward intelligent, multi-modal frameworks that can automate and improve diagnostic precision. This research aligns with that direction by proposing a robust multiclass classification model that leverages both ECG and PCG data for improved CVD prediction.

### 2.1 ECG- and PCG-based CVD diagnosis

The cardiovascular system is responsible for transporting oxygen and nutrients in the blood. A heart, circulatory system, and network of blood vessels make up this system. In order to diagnose CVD, specialists in the field, known as cardiologists, listen for the heart’s rhythm and blood flow with a conventional stethoscope and a phonetic cardiogram. A cardiologist will use a stethoscope to detect vibrations caused by the heartbeat and other sounds, such as murmurs, that are recorded for medical diagnostic purposes. These sounds are commonly referred to as PCG signals. In order to help specialists identify CVDs efficiently from PCG signals in the early stages, a method for automatic recognition of HVDs has been developed. Numerous resources are at the disposal of medical professionals to aid in the rapid and accurate diagnosis of CVD in clinical settings. Using supervised and unsupervised recurrent neural network (RNN)-based Bidirectional Long Short-Term Memory (Bi-LSTM) Machine Learning (ML) algorithm, Vinay et al. (2024) proposed work primarily aims to offer an AI-based PCG signal analysis for the automatic and early detection of various cardiac conditions.

An irregular heartbeat is a common cause of cardiovascular disease, the leading contributor to mortality worldwide. The key to preventing deaths is early diagnosis and prompt treatment. Important non-invasive methods for identifying these diseases include ECGs. Cardiology patients can now more easily undergo remote monitoring because of the proliferation of telemedicine. Data transfer efficiency is crucial for telemedicine sensors due to their limited bandwidth and battery life. With the goal of improving performance, reducing energy consumption, and maintaining diagnostic accuracy in telemedicine, Aggarwal et al. (2021) presented a Latent Space Classification System (LSCS) that compresses electrocardiogram (ECG) signals into smaller dimensions. Using FLOPs, inference time, and transmission size, the study examines energy usage in order to overcome sensor constraints in different feature extraction strategies. In order to compress ECG signals, the suggested LSCS uses a deep convolutional autoencoder that was trained on the MIT-BIH arrhythmia database.

A major cause of death, CVDs have recently emerged as a major physiological condition. Protecting patients from further injury requires accurate and timely detection of heart disease. A number of recent studies have demonstrated the great utility of data-driven methods, such as DL and ML techniques, in the medical profession for the rapid and precise diagnosis of cardiac illness. In contrast, feature engineering is essential for statistical learning and conventional ML methods in order to produce data features that are both robust and effective for use in prediction models. Both procedures present significant obstacles when dealing with big, complicated data sets. On the other hand, DL approaches can automatically learn features from data, and they excel at handling complex and huge datasets, even more so than ML models. In order to overcome the obstacles caused by imbalanced data, Khan et al. (2024) aimed to accurately forecast CVDs by taking the patient’s health and socioeconomic status into account. When it comes to data balancing, the author employed the Adaptive Synthetic Sampling Technique, and for feature selection, the Point Biserial Correlation Coefficient.

The goal of applying machine learning to patient data in illness care is to reap the many significant benefits that come with doing so. However, there are a number of obstacles that arise from the very nature of patient data. In contrast to uncommon or specific cases, which often have small patient sizes and episodic observations, prevalent cases collect a lot of longitudinal data because of the number of patients they follow up with and how consistently they do so. However, longitudinal laboratory data are notorious for being irregular, their temporality, absenteeism, and sparseness. Chen et al. (2024) used self-supervised learning (SSL) to train a GLP model that tracks the overall development of six common laboratory markers in common cardiovascular cases. The goal was to use this knowledge to help detect specific cardiovascular events. Approach and steps: In order to improve SSL’s performance, GLP used a two-stage training method that made use of the data included within interpolated sets. Transferring it to target vessel revascularization (TVR) detection follows GLP pre training. Pure SSL’s performance was enhanced by the suggested two-stage training, and GLP’s transferability was noticeable.

In recent years, the field of CVD detection has seen significant advancements, with researchers exploring innovative approaches to enhance both accuracy and efficiency. Below is a comprehensive analysis of key research contributions that focus on the recognition and classification of cardiac abnormalities through the integration of synchronized ECG and PCG signals, as well as the utilization of DL and wearable technology. Li P. et al. (2021) offered a holistic approach to CVD prediction by integrating multi-modal features, enhancing the predictive power of their model. This study demonstrated promising results, showcasing the potential of combining different types of features for more accurate predictions. However, further analysis of the scalability and generalizability of the proposed model could strengthen the paper’s contributions. Khan et al. (2022) focused on the application of artificial neural networks and spectral features. The integration of spectral features provided valuable insights into the frequency domain, potentially capturing subtle patterns indicative of cardiovascular conditions. Although the methodology was innovative, a deeper exploration of the network architecture and the interpretability of the spectral features could enhance the paper’s impact. Jyothi and Pradeepini (2024) introduced a detection system utilizing both PCG and ECG signals with a machine learning classifier. The inclusion of both signals contributed to a more comprehensive understanding of cardiac health, and the hybrid classifier employed by the authors resulted in improved prediction accuracy. However, a more detailed discussion of the model’s interpretability and potential limitations would further strengthen the paper’s contribution. Do et al. (2023) explored nondestructive detection methods by examining the coupling of PCG and ECG signals to assess coronary artery disease (CAD) stenosis severity. This innovative approach provided a non-invasive means of evaluating cardiovascular health, demonstrating the potential of synchronized signals in assessing disease severity. Khan et al. (2021) proposed a classification system for multi-class CVD using an ensemble method. The use of ensemble techniques enhanced the model’s predictive performance, while impulsive domain analysis offered valuable insights into transient phenomena. The paper’s comprehensive approach was a strength, though a more extensive discussion on computational efficiency and potential deployment challenges would enrich the contribution. Chakir et al. (2020) addressed challenges in cardiac abnormality recognition by synchronizing PCG and ECG signals. The integration of these vital cardiac signals provided a holistic view of heart function, and the authors’ novel methods for signal synchronization yielded promising results in accurately identifying cardiac abnormalities. This collaborative analysis of ECG and PCG signals holds great potential for enhancing diagnostic precision. Li H. et al. (2021) focused on the integration of multi-domain deep features, proposing an advanced method for detecting CAD. By leveraging DL techniques, the authors demonstrated the effectiveness of combining information from both PCG and ECG signals. The integration of multi-domain features enhanced the model’s ability to discern subtle patterns associated with CAD, highlighting the potential for more accurate and reliable disease detection. Wang et al. (2022) designed a wearable monitoring tool for CVD detection. The integration of DL algorithms enabled continuous monitoring, facilitating the early detection of cardiac abnormalities. The wearable aspect of the device enhanced patient compliance and provided a convenient solution for long-term monitoring. The findings underscored the feasibility of employing wearable technology to improve the efficiency of CVD detection. Iqtidar et al. (2021) focused on signal analysis, utilizing mel frequency cepstral coefficients (MFCC) and one-dimensional memory patterns for CVD detection. The incorporation of advanced signal processing techniques enhanced the model’s discriminatory power. The use of PCG signals, in conjunction with these methods, demonstrated the potential for precise and automated classification of cardiac conditions. In conclusion, these research papers collectively contribute to the evolving landscape of CVD detection by presenting innovative approaches that leverage synchronized ECG and PCG signals, multidomain deep features, wearable technology, and advanced signal processing techniques. The integration of these methodologies holds promise for improving the accuracy, efficiency, and accessibility of CVD diagnosis, ultimately benefiting both healthcare professionals and patients.

### 2.2 ECG-based CVD diagnosis

In recent years, the field of CVD detection and management has seen significant advancements through the integration of ECG data and ML models. Haleem et al. (2021) introduced a time-adaptive approach to ECG-driven CVD detection, emphasizing the dynamic nature of ECG signals. Their research demonstrated how real-time adjustments to ECG signal analysis can significantly enhance the accuracy of CVD detection. The adaptive algorithm they presented promises to be a more responsive and precise diagnostic tool, facilitating timely interventions for patients at risk of CVD. Dai et al. (2021) contributed to this evolving field by developing an automatic screening tool for CVD based on convolutional neural networks (CNNs). Their research highlighted the potential of DL models in analyzing various intervals of ECG signals to capture intricate patterns that may indicate the presence of CVD. The robust and reliable screening mechanism developed in this study underscores the transformative power of CNNs in revolutionizing CVD diagnostics. Boonstra et al. (2023) explored the intersection of ECG methodologies and clinical practice, focusing on how ECG data can be utilized for managing CVD. This research provided valuable insights into the potential of ECG data to support personalized treatment strategies. By enhancing clinical decision-making, this study contributes to the growing field of precision medicine in cardiology, offering new avenues for individualized patient care. Angelaki et al. (2021) took a different approach by leveraging ML techniques for the early detection of CVD. Their innovative research demonstrated how ML models could be used to identify subtle structural anomalies in ECG data, offering a preventive strategy for individuals at risk of developing CVD. This approach highlights the potential of ECG-driven ML models to serve as early warning systems, enabling proactive healthcare interventions. Finally, Malakouti (2023) presented a comprehensive heart disease classification model based on ECG signals and ML techniques. Their research showcased the versatility of ML models in categorizing various cardiac conditions, providing a valuable tool for healthcare professionals to streamline diagnosis and treatment plans. This study underscores the role of ML in enhancing the efficiency and accuracy of cardiac care. In conclusion, these five research papers collectively underscore the transformative impact of ECG-driven methodologies and ML models in advancing CVD detection and management. The integration of real-time adaptive algorithms, convolutional neural networks, and personalized medicine approaches highlights the promise of these technologies in shaping the future of cardiology. As the field progresses, these studies pave the way for more sophisticated, accurate, and patient-centric solutions in cardiovascular healthcare.

### 2.3 PCG-based CVD diagnosis

In recent years, the field of cardiac disease diagnosis has witnessed significant progress, largely due to the integration of ML methods into the analysis of PCG signals. This section reviews no table research papers that contribute to this rapidly evolving field, highlighting the potential of ML in revolutionizing the detection and classification of various cardiac disorders. Yadav et al. (2020) explored the application of ML algorithms for classifying cardiac diseases based on PCG recordings. Their study demonstrated the efficacy of these methods in accurately identifying and categorizing different cardiac conditions, thereby contributing to the development of more efficient diagnostic tools. Baghel et al. (2020) focused on leveraging CNNs to automate the diagnosis of multiple cardiac diseases using PCG signals. The utilization of CNNs showcases the potential for DL architectures to capture intricate patterns within heart sounds, leading to improved diagnostic accuracy. Tuncer et al. (2021) employed pattern recognition techniques to automate the detection of cardiac abnormalities from PCG signals. Their approach illustrates the diversity of methods within the field, combining traditional signal processing with modern ML techniques to enhance diagnostic capabilities. Talal et al. (2023) concentrated on classifying a diverse set of heart disorders, emphasizing the versatility of ML models in handling a wide range of abnormalities in PCG signals. This study underscores the importance of comprehensive models that can accurately diagnose various cardiac conditions. Shuvo et al. (2021) introduced the CardioXNet framework, a lightweight DL approach tailored for CVD classification. Their research highlights the need for models that balance accuracy and computational efficiency, ad-dressing practical considerations for real-world applications. Li et al. (2020) proposed a fusion method that combines multiple field features with DL to enhance coronary artery disease detection. The integration of diverse features reflects a holistic approach toward improving the robustness and accuracy of diagnostic systems. In conclusion, these research papers collectively contribute to advancing cardiac disease diagnosis through the integration of ML and DL techniques. The diverse methodologies presented in these studies underscore the richness of approaches in this field, promising a future where accurate and efficient cardiac disease diagnosis becomes more accessible to clinicians and patients alike. In summary, existing studies have explored various approaches for CVD detection using either ECG or PCG signals, as well as deep learning and machine learning techniques. However, most of these methods are limited by single-modality analysis, lack of robust preprocessing, or insufficient feature extraction mechanisms. Few works fully exploit synchronized ECG and PCG data with advanced attention-based architectures for multiclass classification. Addressing these gaps, our proposed work introduces a novel multi-modality framework incorporating SST-PNet, a tripattern attention mechanism, and a self-supervised EnDe-CNN classifier. This integrated approach significantly enhances the diagnostic performance and contributes a comprehensive, scalable model to the current body of knowledge in CVD detection. Table 1 highlights recent research on CVD detection using diverse approaches.

**TABLE 1:** Research Gaps of state of arts.

Study	Approach	Key contributions	Limitations
Li et al. (2021a)	Multi-modal feature integration	Enhanced predictive power for CVD through integration of various features	Scalability and generalizability need further exploration
Khan et al. (2022), Wang et al. (2022)	Artificial neural networks and spectral features	Used frequency features for inputs into deep learning, improving CVD detection accuracy	Black-box explanation and interpretability are difficult
Jyothi and Pradeepini (2024)	Hybrid classifier with PCG and ECG signals	Achieved improved prediction accuracy by using both signals	Requires more annotated datasets; sensor integration limitations
Do et al. (2023)	Non-destructive detection with PCG and ECG coupling	Innovative non-invasive approach for assessing CAD severity	Limited diagnosis and broader applicability need validation
Khan et al. (2021)	Ensemble methods for multiclass CVD classification	Enhanced predictive performance and provided insights into transient phenomena	Needs more contextual efficiency and deployment challenges
Chakir et al. (2020)	PCG and ECG signal synchronization	Novel methods for signal synchronization, improving accuracy in cardiac abnormality recognition	Engineering synchronization of the two systems affects robustness
Li et al. (2021b)	Multidomain deep features	Demonstrated effective CVD detection by integrating multidomain features	Computational complexity and real-time applicability
Wang et al. (2022)	Wearable monitoring tool for CVD detection	Enabled continuous monitoring through DL algorithms, enhancing early detection	Device performance issues and patient adherence are challenges
Iqtidar et al. (2021)	MFCC and ID memory patterns for CVD detection	Improved model’s discriminatory power through advanced signal processing techniques	Requires validation on diverse datasets
Haleem et al. (2021)	Time-adaptive ECG-driven CVD detection	Improved accuracy through real-time adaptive signal analysis	Needs further validation in clinical settings
Dai et al. (2021)	CNN-based automatic screening tool	Highlighted DL’s potential in expanding multiple ECG patterns for CVD detection	Limited generalizability to diverse populations
Boonstra et al. (2023)	ECG methodologies for clinical practice	Forward screening into ML for personalized treatment strategies	Needs broader clinical validation
Angelaki et al. (2021)	ML for early detection of CVD	Demonstrated ML’s power in early CVD detection through ECG waveforms	Requires more research with real-world data
Malakouti (2023)	ML for heart disease diagnosis	Showcased ML’s versatility in addressing various cardiac patterns	Needs further exploration on scalability and validation
Yadav et al. (2020)	ML algorithms for PCG-ECG fusion	Achieved accurate classification using multimodal synchronization	Limited by the need for high-quality synchronized input
Baghel et al. (2020)	CNNs for PCG-based diagnosis	Improved the diagnostic accuracy by capturing intricate patterns in heart sounds	Requires validation across more diverse patient groups
Tuncer et al. (2021)	Pattern recognition for PCG signals	Enhanced diagnostic capabilities through traditional signal processing and ML.	Needs further refinement of the pattern recognition methods
Talal et al. (2023)	ML with ECG features	Showcased ML’s versatility in diagnosing various cardiac disorders	Requires further testing in clinical environments to ensure robustness
Shuvo et al. (2021)	CardioXNet for lightweight DL CVD classification	Balanced accuracy and computational efficiency for real world applications	Needs further exploration of its performance in resource-limited settings
Li et al. (2020)	Fusion method with DL for CAD detection	Enhanced diagnostic robustness and accuracy through multi field feature integration	Limited by the need for further testing on different types of CVD.

## 3 Multi-class framework

**FIGURE 1:**
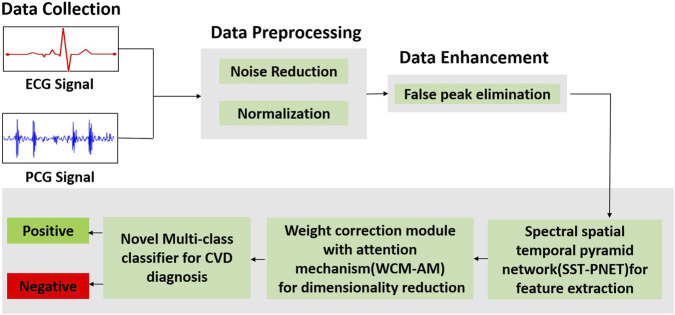
Overall workflow of the proposed framework illustrates the complete pipeline of the proposed multi-class CVD diagnosis system, encompassing multimodal data collection (ECG and PCG), advanced preprocessing and enhancement, followed by feature extraction, attention-based weighting, and final classification into positive or negative CVD cases.

Our proposed multi-class framework integrates the multimodality of ECG and PCG signals for diagnosing CVD. We designed and proposed a multiclass classifier for individual input channels. In this research, a multiclass architecture comprises three modules for feature extraction, feature selection, and classification. Figure 1 illustrates the complete pipeline of the proposed multiclass CVD diagnosis system, encompassing multimodal data collection (ECG and PCG), advanced preprocessing and enhancement, followed by feature extraction, attention-based weighting, and final classification into positive or negative CVD cases.

### 3.1 Data collection

ECG and PCG records stood sourced from 2016 PhysioNet/CinC Challenge, with contributions from various foreign universities to the databases. The dataset, categorized into six subsets labeled training-1 through training-6 is distributed across different establishments. The “training-a” dataset consists of 409 recordings, where 405 records utilize a Welch Allyn Meditron electronic stethoscope with a frequency response range of 25 Hz–40 kHz. Among these, 117 recordings are classified as negative, representing the standard control set, while the remaining 288 are from patients diagnosed with mild aortic disease (AD), mitral valve prolapse (MVP), or other pathological diseases (MPC) and are classified as positive. The PCG and ECG signals in these recordings undergo resampling to 2000 Hz. Some recordings, or parts thereof, exhibit visual noise, making interpretation challenging. Consistent with previous research, 17 recordings containing noise are manually removed to mitigate potential bias. Table 2 summarizes the dataset. The values (Mean, Min, SD, Median, and Max) represent the statistical distribution of the recording duration (in seconds) across subjects. It is important to take that 388 recordings are divided into training and validation datasets before completing the data expansion process.

**TABLE 2:** Description of the dataset.

Classes	Subjects	Time length (s)				
		Mean	Min	SD	Median	Max
Positive	273	31.65	11.72	5.87	34.64	35.92
Negative	115	31.74	8.98	4.22	30.58	35.92

The records vary significantly in length, and to provide an ample quantity of data for a deep neural network, we employ a sliding window approach to augment the dataset. Specifically, we divide the lengthy raw signals into short recordings with an 8-s frame. The window stride for positive recordings is set at 8 s, while for negative recordings, it is 3 s, ensuring a balanced distribution of positive and negative recordings. Subsequently, through these segmentations, we adjust the ratio from 2.4:1 (273:115) to approximately 1:1. Table 3 presents the dataset composition, consisting of 1008 positive and 967 negative samples, each with a duration of 8 s.

**TABLE 3:** Data descriptio**n**.

Classes	Subjects	Time length (s)
Positive	1008	8
Negative	967	8

### 3.2 Data preprocessing

#### 3.2.1 Noise reduction

ECG and PCG signals are prone to various types of noise such as baseline wander (low-frequency drift due to respiration or electrode movement), powerline interference (50/60 Hz), and motion artifacts caused by patient activity. To address these challenges, Wiener filtering was employed for noise reduction. The Wiener filter is particularly suitable for ECG/PCG denoising because it minimizes the mean square error (MSE) between the desired clean signal and the estimated signal, while considering both the signal and noise as stochastic processes. This makes it highly effective for biomedical signals where the spectral overlap between noise and signal is significant.

The Wiener filter adapts its coefficients $\Psi_{k}$ based on the estimated spectral characteristics of the noisy signal. For an input signal x(n) consisting of the true signal ϱ(n) and noise v(n) , as represented in Equation 1.

$$x\left(n\right)=\varrho \left(n\right)+v\left(n\right),$$ (1)

The output signal λ(n) provides an estimate of ϱ(n) . The error signal ε(n) is given by as represented in Equation 2.

$$\varepsilon \left(n\right)=\lambda \left(n\right)-\varrho \left(n\right),$$ (2)

and the Wiener filter seeks to minimize the mean squared error, as represented in Equation 3.

$$\varepsilon =\min\left(E\left[\varepsilon^{2}\left(n\right)\right]\right).$$ (3)

The discrete Wiener filter is expressed as represented in Equation 4.

$$\lambda \left(n\right)=\overset{N-1}{\underset{ℵ=0}{\sum }}\Psi_{ℵ}\left(d\left(n-K\right)\cdot v\left(n-ℵ\right)\right),$$ (4)

where the filter coefficients $\Psi_{ℵ}$ are iteratively adapted. The Wiener–Hopf equation provides the condition for optimal weights as represented in Equation 5:

$$\overset{p-1}{\underset{l=0}{\sum }}\Psi_{ol}\;r_{xx}\left(k-l\right)=r_{x\varrho }\left(-l\right),$$ (5)

where $\Psi_{o0},\Psi_{o1},\ldots ,\Psi_{op-1}$ denote the optimum tap weights of the filter, $r_{xx}$ represents the autocorrelation function of x(n) , and $r_{x\varrho }$ denotes the cross-correlation function between x(n) and ϱ(n) . In the proposed multiclass model, the filter order and window length were empirically determined through preliminary trials to maximize noise suppression while preserving the morphological details of the ECG and PCG signals. This ensured that clinically relevant features (such as QRS complexes in ECG and systolic/diastolic components in PCG)were retained after filtering.

#### 3.2.2 Normalization

After noise reduction, the min–max normalization technique of data normalization is performed in which linear transformation is executed on noise reduced data. The value of maximum and minimum from data is increased, and the individual value is changed as per the following formula,

$$\Delta ´=\frac{\Delta -\min\left(∄\right)}{\max\left(∄\right)-\min\left(∄\right)}\left(new_{-}\max\left(∄\right)-new_{-}\min\left(∄\right)\right)+new_{\min\left(∄\right)}$$ (6)

where ∄ denoted as attribute data, $\max\left(∄\right)$ denotes the maximum absolute value, and $\min\left(∄\right)$ denotes the minimum absolute value. Δ´ and Δ are the new value and old values of individual data entry, respectively. Furthermore, $new_{-}\min\left(∄\right)$ and $new_{-}\max\left(∄\right)$ denote the parameters of maximum and minimum value of range, respectively as represented in Equation 6.

### 3.3 Data enhancement

#### 3.3.1 False peak elimination

Likewise, for the proficient extraction of characteristics from the ECG and PCG signals, we eliminate inaccurate peaks and reconstruct it. Within ECG and PCG signal processing, the process of identifying and removing or reducing misleading or inaccurate peaks in the ECG and PCG signal is termed false peak elimination. The elimination of false peaks in ECG and PCG signal segmentation involves scrutinizing peak-to-peak intervals through statistical assessment of false peaks. The subsequent features, precisely defined as follows, were statistically identified and employed for false peak elimination using an adaptive threshold along the amplitude axis.

1. Frequency Analysis: Power Spectrum

The identification and separation of frequency components associated with genuine brain activity from noise can be achieved by analyzing the power spectrum of ECG and PCG recordings.

2. Time-related Features: Temporal Constancy and Variability

Genuine ECG and PCG signals exhibit significant temporal constancy. Artifacts may manifest as sudden, abrupt changes or spikes. Measuring the variability of the ECG and PCG signal over time assists in pinpointing segments likely to contain artefacts.

3. Spatial Characteristics: Consistency and Channel Correlation

ECG and PCG data acquired from multiple electrodes should demonstrate spatial consistency. Inconsistencies in spatial patterns could indicate artifacts. Evaluating the correlation between ECG and PCG channels aids in identifying anomalous patterns associated with artifacts.

4. Amplitude and Duration of Waveform Morphology: Distinctive Characteristics

Authentic ECG and PCG signals often showcase distinctive amplitudes and durations. Anomalies may be signaled by unusual spikes in amplitude or duration. Analyzing the morphology and shape of ECG and PCG waves helps identify abnormalities related to artifacts.

5. Statistical Measures: Moments and Anomaly Detection

Calculating statistical moments (mean, variance, skewness, and kurtosis) of ECG and PCG segments assists in identifying anomalous patterns linked to artifacts. Anomaly detection through statistical techniques aids in the effective elimination of false peaks.

### 3.4 CVD diagnosis

Next, we fed our processed data into multi-class architecture which consists of three modules as shown in Figure 1: (i) SST-PNet to extract appropriate features; (ii) WCM-AM which provide significant weight for optimal features by means of feature selection; (iii) Novel Multi-class Classifier is introduced for classifying the several CVDs into multiple classes.

#### 3.4.1 Spectral Spatial Temporal Pyramid Network (SST-PNet)

**FIGURE 2:**
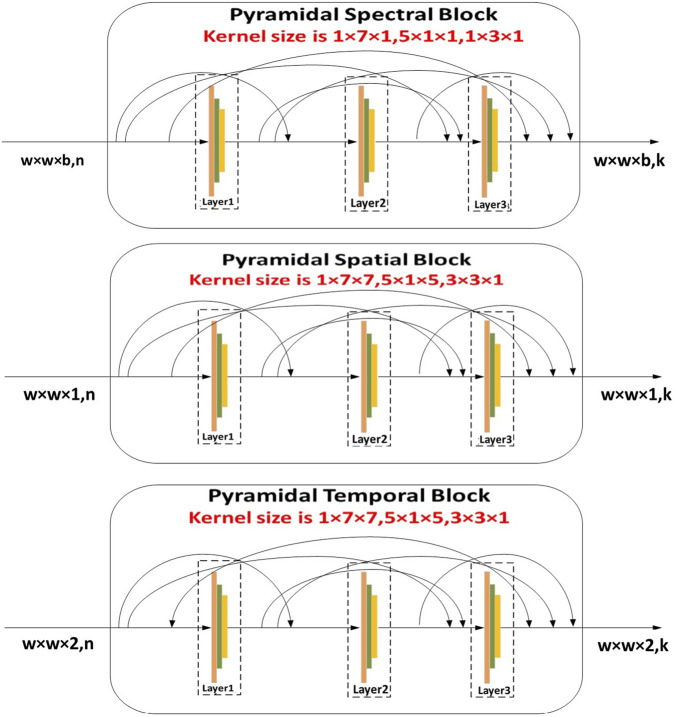
Illustration of SST-PNet depicts the structure of the SST-PNet, which consists of three parallel branches, namely, pyramidal spectral, spatial, and temporal blocks, each applying multi-scale 3D convolutions to capture hierarchical features across different dimensions of ECG and PCG data.

Once both ECG and PCG signals are preprocessed and enhanced, we fed the data into a multi-class model. Here, primarily SST-PNet is adapted for feature extraction for CVD diagnosis. SST-PNet consists of spectral, spatial, and temporal branches in which pyramidal convolution is encompassed, as shown in Figure 2. Initially, the pyramidal blocks utilized in three branches are detailed as follows,

1. Spectral Branch- Initial Layer: Typically, the initial step in feature modification involves the application of a 3D convolutional layer, aimed at reducing the computational burden along the spectral dimension. Subsequently, a pyramidal spectral block is appended. In Figure 2, each layer within the pyramidal convolution consists of three 3D convolution procedures with progressively decreasing spectral dimension levels. For each layer, the kernel sizes of the 3D convolution operations are sequentially set as 1 × 1 × 7, 1 × 1 × 5, and 1 × 1 × 3.
2. Construction: Subsequent to each convolution, a batch normalization (BN) layer is introduced for regularization, followed by the application of the Mish activation function. This combination aids in learning a nonlinear representation, thereby enhancing the network’s power and speed of convergence. Each layer maintains a consistent number of output channels, customizable as γ´ . The final output number for the block can be expressed as follows:

γ=η+3∗γ´ (7)

In the equation, γ represents the actual number of 3D convolution kernels, and n is the number of output channels from the preceding 3D convolution layer. It is noteworthy that the investigation primarily focuses on spectrum information, given that the only variable in these convolution kernels is their spectral dimension, which is never equal to 1.

• Spatial Branch: Construction: This branch also utilizes a pyramidal structure, with convolution kernels varying in spatial dimensions while maintaining consistency in the spectral dimension. A 3D convolutional layer is applied initially, followed by the pyramidal spatial block, which includes layers with batch normalization and Mish activation functions.

• Temporal Branch: Construction: Similar to the spatial branch, the temporal branch involves pyramidal convolution with variable kernel sizes in the temporal dimension. A 3D convolutional layer is applied before the pyramidal temporal block, with each layer featuring batch normalization and Mish activation functions.

Each branch of SST-PNet is designed to extract different types of features from the multimodality input data, leveraging interspatial linkages and dimension-specific kernels to enhance feature representation and improve the diagnostic accuracy.

**FIGURE 3:**
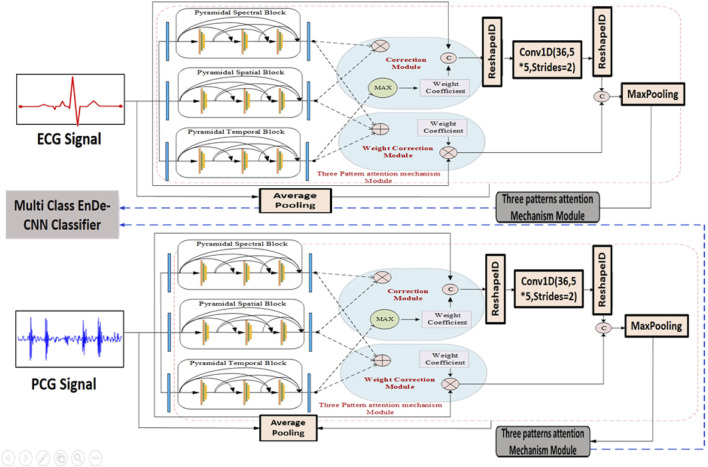
Architecture of SST-PNet and WCAM with Three-pattern Attention Mechanism illustrates the dual-stream processing of ECG and PCG signals using the SST-PNet for feature extraction, followed by a WCAM that adaptively refines features across spectral, spatial, and temporal domains before classification by the multi-class EnDe-CNN.

Likewise, the construction of the pyramidal spatial and temporal blocks leverages interspatial linkages within feature maps, similar to the pyramidal spectral block. As shown in Figure 2, the kernel size in the spatial and temporal blocks varies along the spatial dimension while remaining fixed in the spectral dimension. Moreover, a 3D convolution layer is applied before compressing the spectral and temporal dimensions. Each layer within these blocks includes a 3D convolutional layer, followed by batch normalization and a Mish activation function. The relationship between the input and output of the pyramidal spatial and temporal blocks is defined in Equation 7. Furthermore, Figure 3 demonstrates how the pyramidal spectral, spatial, and temporal blocks are integrated with a three-pattern attention mechanism and weight correction modules to extract and refine features from ECG and PCG signals before classification.

#### 3.4.2 Weight correction module with the Tri-Pattern Attention Mechanism

The WCM-AM was implemented by integrating a three-pattern attention mechanism within a weight correction framework to address imbalances in feature importance. This attention mechanism selectively focuses on critical regions of the input data, enhancing the model’s ability to identify relevant features for accurate classification. The WCM-AM was trained using a combination of supervised learning techniques, with attention weights adjusted based on gradients to optimize feature selection. During training, the model iteratively refined its attention patterns and weight corrections to improve classification performance and reduce misclassification rates.

1. Three-Pattern Attention:

Here, we proposed that the Tri-Pattern Attention Mechanism (TPAM) acquires attention weights in spectral, spatial, and temporal directions. Assume the extracted feature map from prior layers as $\beta_{i,j}^{\mathrm{spe}}\in R^{H*W}$ , where H and W denote height and width of feature maps, respectively, and $\beta_{i,j}^{\mathrm{spe}}$ into spectral attention module to obtain attention weight as represented in Equation 8. The following is the mathematically determined as

$$Att_{\mathrm{spe}}=\frac{exp\left(W_{\mathrm{spe}}\beta_{i,j}^{h}+b_{\mathrm{spe}}\right)}{\Sigma_{i,j}exp\left(W_{\mathrm{spe}}\beta_{i,j}^{h}+b_{\mathrm{spe}}\right)}$$ (8)

From the abovementioned equation, $W_{\mathrm{spe}}$ and $b_{\mathrm{spe}}$ are denoted as weight parameters of the dense layer, and $Att_{\mathrm{spe}}$ represents the attention coefficient of the spectral pattern. In addition, for the attention mechanism of temporal, we transpose the matrix of feature map to acquire the feature map of the temporal pattern. The procedure is mathematically expressed as

$$\beta_{i,j}^{\mathrm{tem}}={\left(\beta_{i,j}^{\mathrm{tem}}\right)}^{T}$$ (9)

Where $\beta_{i,j}^{\mathrm{tem}}$ represents the feature map with temporal attention coefficient as represented in Equation 9. The input of the temporal attention module is similar to obtain the attention weight of the temporal pattern as represented in Equation 10. The weight coefficient is determined as

$$Att_{\mathrm{tem}}=\frac{exp\left(W_{\mathrm{tem}}\beta_{i,j}+b_{\mathrm{tem}}\right)}{\Sigma_{ij}exp\left(W_{\mathrm{tem}}\beta_{i,j}+b_{\mathrm{tem}}\right)}$$ (10)

For the attention mechanism of the spatial pattern, we mainly focus on weight distribution among feature maps to further acquire spatial features. Let the feature of input in the attention module of the spatial pattern be ${\left(\beta_{i,j}^{\mathrm{spa}}\right)}^{k}\in R^{H*W*K},\left(\beta_{i,j}^{\mathrm{spa}}\right)=\left[{\left(\beta_{i,j}^{\mathrm{spa}}\right)}^{1},{\left(\beta_{i,j}^{\mathrm{spa}}\right)}^{2},\ldots .,{\left(\beta_{i,j}^{\mathrm{spa}}\right)}^{k}\right]$ , here k indicates the index of the feature map, and the following equation is utilized to determine the spatial pattern attention weight coefficient,

$$Att_{\mathrm{spa}}=\frac{exp\left(W_{\mathrm{spa}}{\left(\beta_{i,j}^{\mathrm{spa}}\right)}^{k}+b_{\mathrm{spa}}\right)}{\Sigma_{ij}exp\left(W_{\mathrm{spa}}{\left(\beta_{i,j}^{\mathrm{spa}}\right)}^{k}+b_{\mathrm{spa}}\right)}$$ (11)

Here, $Att_{\mathrm{spa}}$ denotes the spatial pattern attention coefficient as represented in Equation 11.

2. Weight Correction Strategy: We proposed two methods, namely, weight maximization and addition, to address the weight coefficients corresponding to the three attention directions acquired in the preceding stage. In the weight-maximization approach, we doubled the weight coefficients associated with horizontal and vertical attention based on our previous findings, showcasing its remarkable performance through empirical trials. In pursuit of enhanced spatial characteristics, we introduced a weight addition approach in the present study. Specifically, this approach involves adding the attention weight coefficient of the spatial direction to the existing foundation. The resulting coefficient in the weight addition strategy’s output can be obtained using the following formula:

$$Att_{\mathrm{add}}=\left(Att_{\mathrm{spe}}+Att_{\mathrm{tem}}+Att_{\mathrm{spa}}\right)$$ (12)

From the Equation 12, $Att_{\mathrm{add}}$ denotes the weight addition mechanism’s output coefficient. For the mechanism of maximum weight, we consider the principal one of weight coefficients, and the equation is as follows:

$$Att_{\max}=\max\left[\left(Att_{\mathrm{spe}}⊗Att_{\mathrm{tem}}\right)Att_{\mathrm{spa}}\right]$$ (13)

Where $Att_{\mathrm{add}}$ denotes the weight maximization mechanism’s output coefficient. Assume $Att_{\mathrm{add}}$ concatenate the feature map of input $\beta_{i,j}^{k}$ to acquire the weight addition mechanism’s output feature map. Moreover, $Att_{\max}$ as represented in Equation 13 multiply the feature map of input $\beta_{i,j}^{k}$ and done Conv3D as represented in Equation 14 to acquire the weight addition mechanism’s output feature map. Finally, we concatenate the three-pattern attention mechanism as represented in Equations 15, 16.

$$I_{\mathrm{add}}=Conv3D\left(Att_{\mathrm{add}}⊗\beta_{i,j}^{k}\right)$$ (14)

$$I_{\max}=concat\left[I_{\max},\beta_{i,j}^{k}\right]$$ (15)

$$I_{\mathrm{TPAM}}=max_{\mathrm{pool}}\left\{concat\left[I_{\max},I_{\mathrm{add}}\right]\right\}$$ (16)

Where $I_{\mathrm{TPAM}}$ denotes the output of the final three-pattern attention mechanism, concat () is the operation of feature fusion and concatenation, and $max_{\mathrm{pool}}$ is the max pooling.

To make feature weighting more effective and easier to understand, the MCC-CVD model architecture incorporates the TPAM and the Weight Correction Strategy. TPAM is built to take a look at input information from three different angles, each emphasizing a different level of semantic relevance: global, local, and residual. This allows it to extract diverse attention patterns. An initial feature significance map is created by aggregating the outputs of the three attention pathways. This map reflects the multiscale dependencies across the input attributes. The next step, rather than a substitute, is to use the Weight Correction Strategy sequentially. The attention-derived weights are fine-tuned in this module by means of two procedures: weight addition and weight maximization. As a sort of soft selection, weight maximization in the first step amplifies the most informative traits, which are those that are consistently highlighted across TPAM branches. By including contextual information from nearby characteristics or states (in time-dependent data), the second stage of weight addition prevents the suppression of any important but underrepresented patterns. The maximal weights serve as a foundation for addition rather than individual strategy routes in this sequential application of stages. TPAM and the Weight Correction Strategy produce detailed attention signals from several angles, and the latter acts as a post-attention calibration mechanism to additionally enhance the importance of features. Classification accuracy and interpretability in cardiovascular disease diagnosis are both enhanced by this two-stage refinement, which allows the model to zero down on clinically relevant variables like age, blood pressure, and comorbidity indicators. To construct spectral features, the MCC-CVD framework examines the distributional patterns of clinical variables. Each feature is then represented in a modified space that emphasizes the links between its statistical frequency and value range. By seeing the feature space as a structured relationship graph, with edges representing correlations or co-occurrence strengths, we may model the interdependencies between clinical variables and obtain spatial features. Utilizing recurrent encoders, temporal features are retrieved from medical records that contain sequential measures in order to capture patterns that evolve over time. After feature extraction, a hierarchical attention mechanism is used to fuse the three domains of features. First, the TPAM assigns weights that are specific to each domain. Then, the Weight Correction Strategy refines and combines the features into a single representation for classification. In MCC-CVD, the pyramid network is built to gradually abstract feature representations across several levels of resolution. A three-tiered pyramid structure is employed to process the input fused feature map, with each tier decreasing the dimensionality and increasing the learnt patterns’ abstraction level. There are three steps to the process: preserving local features at a high resolution, capturing correlations at an intermediate level, and finally encoding global semantic patterns that are relevant to cardiovascular risk assessment. Convolutional layers, batch normalization, and activation functions make up each step, and skip connections permit fine-grained information to be preserved. A balance between computing efficiency and representational richness is maintained by fixing the pyramid’s depth at three hierarchical levels. MCC-CVD’s classification module uses a fully connected neural network and a Softmax output layer to forecast probabilities across several classes. Prior to being passed through two dense layers with ReLU activation, the fused and pyramid-processed features undergo flattening. This procedure maps the high-dimensional feature space onto a lower-dimensional decision space. To avoid overfitting, regularization with dropout layers is used. Last but not least, the Softmax layer generates probability distributions for each cardiovascular disease category; the class label is associated with the probability that the model used to make its prediction. Although there is only one deep classifier studied, it can be easily extended to include more in future work to create an ensemble that leverages the combined outputs of several classifiers to increase resilience.

#### 3.4.3 Self-supervised multi-class classifier

**FIGURE 4:**
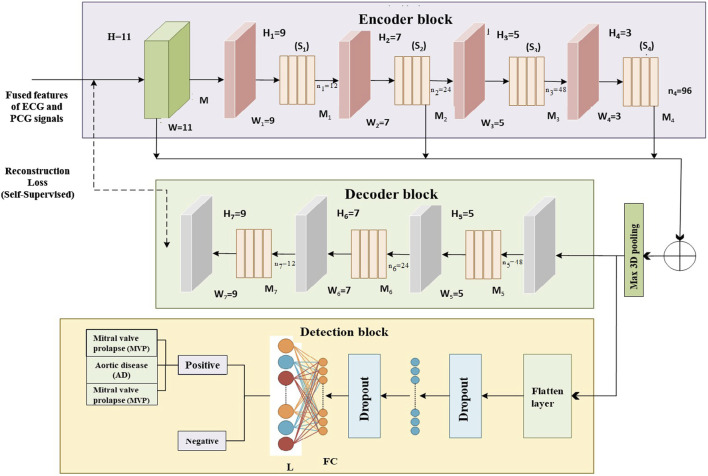
Proposed multi-class EnDe-CNN Classifier presents the architecture of the multi-class EnDe-CNN, where fused ECG and PCG features are encoded and decoded through 3D convolutional layers in a self-supervised fashion, followed by a fully connected detection block that performs multiclass classification of CVD types such as MVP and AD with enhanced robustness via dropout and feature flattening.

The proposed multi-class model, known as EnDe-CNN as shown in Figure 4, presents the architecture of the multi-class EnDe-CNN, where fused ECG and PCG features are encoded and decoded through 3D convolutional layers in a self-supervised fashion, followed by a fully connected detection block that performs multiclass classification of CVD types such as MVP and AD with enhanced robustness via dropout and feature flattening. We utilized aggregated network information as input. Furthermore, the encoder–decoder block within EnDe-CNN contributes to minimizing errors during feature learning, facilitating the acquisition of more discriminative characteristics related to signals. A comprehensive explanation of each block and module in EnDe-CNN is given as follows.

1. Encoder Block: Upon receiving spectral, spatial, and temporal characteristics of both ECG and PCG signals from the previous module are fused, the encoder module meticulously processes the obtained features in three dimensions, element by element. The key components of the encoder module include the batch normalization layer, convolution layer, and ReLU layer. The formulation for the convolution output at the *j*th layer is provided below.

$$Conv^{\left(j\right)}={\left(ConvKer⊗Z\right)}_{\left(j\right)}+bias$$ (17)

$$M_{j}=\frac{M_{j-1}+2*pad\left[0\right]-\left(ConvKer_{siz\left[0\right]}-1\right)-1}{Str\left[0\right]}+1$$ (18)

In the provided Equation 17, Z denotes the input to the *j*th layer, and ConvKer represents the convolutional kernel. The 3D convolutional process is denoted by ⊗ , and bias represents the offset value. Additionally, it is feasible to set the padding and stride for each dimension to 0 and 1, respectively as represented in Equation 18. The batch normalization layer within the encoder module, expressed below, serves the purpose of mitigating undesirable covariate shifts.

$${Op}_{\text{Z}}=\frac{Y_{\text{z}}-\varphi^{\left(Fm\right)}\left[\eta_{\text{z}}\right]}{\sqrt{{\mathrm{Var}}^{\left(Fm\right)}\left[N_{\text{z}}\right]}+3}*\alpha +\sigma ,\omega =1,2,\ldots ,T$$ (19)

The symbols $F_{m}$ and $Y_{\text{z}}$ in the above Equation 19 represent the feature maps and convolution minibatch, respectively. Symbols $\varphi^{\left(Fm\right)}\left[Y_{\text{z}}\right]$ and $Var^{\left(Fm\right)}\left[Y_{\text{z}}\right]$ denote the mean and standard deviation for each feature value, respectively. Hyperparameters α and σ , ω are set to 1 $e^{-4}$ . The ReLU activation is employed to facilitate the convergence of training operations in the encoder network. The multilayer feature concatenation block amalgamates the processed features of the encoder at various depths. The characteristics, combined into a single matrix M, are represented as $agg_{Y}\left(j\right)=\left(j\to z_{1},z_{2},z_{3}\right)$ . The amalgamation process can be described as follows:

$$D=\left[agg_{Y_{\text{z}}},agg_{Y_{z^{2}}},agg_{Y_{4}}\right]$$ (20)

Where agg represents the concatenation operation as represented in Equation 20. In addition, the number of training parameters has diminished through employing the 3D max pooling layer.

2. Decoder Block: The encoder architecture, comprising 3D deconvolutional layers, ReLU activation, and batch normalization layers, mirrors the structure of the decoder blocks. The primary purpose of the decoder block is to map lower-dimensional space to a higher-dimensional space. By performing dimensionality conversion, features of diverse sizes can be restored to a uniform size. Let $\left\{agg_{Y_{\text{j}}}\right\}$ denote the feature patches and n represent the weight. The weight is applied to minimize the reconstruction loss and can be articulated as follows:

$$Loss=\frac{1}{k}\overset{k}{\underset{j=1}{\sum }}{\left\|agg_{I_{j}}-f\left(agg_{I_{j}};n\right)\right\|}_{2}^{2}$$ (21)

In the given Equation 21 k stands for the number of 3D feature patches, k, $agg_{I_{j}}$ represents the feature value, and f (.) denotes the encoding and decoding procedures. In summary, the proposed EnDe-CNN achieves self-supervised learning of spectral–spatial–temporal characteristics through these mechanisms.

This research introduces the MCC-CVD model, a unique, multicomponent deep learning architecture specifically built for multiclass categorization of CVD classes using clinical data. A composite structure that incorporates deep neural layers, feature fusion, and optimal decision thresholds to handle the complexity of various cardiovascular risk signals is what makes the suggested method unique. In contrast to traditional models, the MCC-CVD model uses a hierarchical and multiclass method to categorize CVD. It can distinguish between different subtypes of CVDs that share risk factors and symptoms. The study’s hyperparameter optimization mechanism, which uses grid search to adjust the learning rate, dropout, activation functions, and model depth, is one of its main advances. This tackles a major issue with previous research that used static or default parameter settings, that is, how well the model performed and how well it generalized to new data. In addition, our model incorporates robust statistical evaluation to ensure the reliability of the reported results. This includes 10-fold cross-validation, 95% confidence intervals for performance metrics, and significance testing, such as paired t-tests, in contrast to previous approaches that report single-point performance. The use of sensitivity and robustness analysis is another unique feature of this study. We performed a stress analysis of the MCC-CVD model’s performance with different data distributions and parameter shifts to make it more interpretable and to ensure that it can be used in real-world clinical settings with potentially noisy, imbalanced, or incomplete data. Clinical decision support systems that demand comprehensible, explainable results for risk stratification can use this model because it enables feature importance score and adjustable feature weighting. Statistical rigor, an optimization-driven architecture, realistic robustness testing, and a focus on several classes are what set the MCC-CVD model apart from previous efforts. With these improvements, it is now considered a major step forward in the use of machine learning in healthcare, especially in the crucial area of predicting and diagnosing cardiovascular disease.

## 4 Experimental results

### 4.1 Simulation setup

We apply the mentioned methodologies to the respective datasets. The effectiveness of the multimodal approach is illustrated through the prediction of CVD using the trained multi-class model. The proposed framework, developed using MATLAB 7.12, was designed to improve the Parallel-Multi-class framework. Implementation was conducted on a Windows PC with a 1.6 GHz Intel Core i5 processor and 4 GB of RAM. Additionally, we showcase the efficiency of the pyramid network and weight correction strategy into the feature analyzing process. Each case is validated using a five-fold cross-validation, and we conduct ten repetitions for each instance.

### 4.2 Performance metrics

The efficient of the proposed multiclass model is demonstrated by comparing it to accurate CVD detection methods through the computation of various performance metrics, including accuracy, specificity, sensitivity, F1-score, and ROC curve. The mathematical expressions for these metrics are as follows:

$$Accuracy=\frac{\left|\delta_{TP}+\delta_{FP}\right|}{\left|\delta_{TP}+\delta_{TN}+\delta_{FP}+\delta_{FN}\right|}$$ (22)

$$Specificity=\frac{\left|\delta_{TP}\right|}{\left|\delta_{TP}+\delta_{FP}\right|}$$ (23)

$$Sensitivity=\frac{\left|\delta_{TP}\right|}{\left|\delta_{TP}+\delta_{FN}\right|}$$ (24)

$$F1-score=\frac{2*Specificity*Sensitivity}{Specificity+Sensitivity}$$ (25)

In these equations, $\delta_{TP}$ and $\delta_{TN}$ represent true positive and true negative rates, respectively, while $\delta_{FP}$ and $\delta_{FN}$ denote false positive and false negative rates, respectively. These formulas quantitatively assess the performance of the multi-class model in comparison to alternative PD detection strategies.

### 4.3 Training analysis

An organized and repeated tuning procedure was employed to choose the MCC-CVD model’s parameters. Standard procedures used in earlier research on cardiovascular disease prediction informed the selection of these initial values. On the other hand, a systematic grid search was used to investigate different combinations of important hyperparameters in order to guarantee the best model performance. Here is the definition of the search space: The learning rates were set between 0.001 and 0.05, batch sizes were 32, 64, and 128, and the dropout rates were 0.2–0.5. The number of hidden layers was set between 2 and 4. The sigmoid, tanh, and ReLU activation functions were also tested. The ideal setup, which includes a learning rate of 0.005, a batch size of 64, a dropout rate of 0.3, and three hidden layers activated by ReLU, was chosen because it performed exceptionally well on validation data, producing the best F1 score and balanced precision–recall trade-off. A sensitivity analysis was carried out alongside hyperparameter optimization to assess how different parameter changes affected the model’s performance. In order to see changes in accuracy and F1 score, we adjusted some parameters while keeping others constant. The results showed that the model’s behavior was greatly affected by the learning rate and dropout rate, with performance differences of up to ± 4%. However, batch size and hidden layer count were rather un-important. To guarantee stability and generalizability, our results highlight the significance of finetuning key parameters. In summary, this method prevents the use of arbitrary parameter values and instead guarantees that they are robust and based on empirical evidence. The optimization of network parameters is carried out using Adam’s optimizer. The training procedure for multi class of ECG involves a total of 150 epochs. At every 50 epochs, the learning rate is adjusted by multiplying it by 0.1, starting from an initial value of 0.001. Similarly, for the multi class of the PCG training process, a total of 160 epochs are utilized. The learning rate increases by 0.1 every 80 epochs, commencing from an initial value of 0.001.

### 4.4 Analysis of feature extraction

**FIGURE 5:**
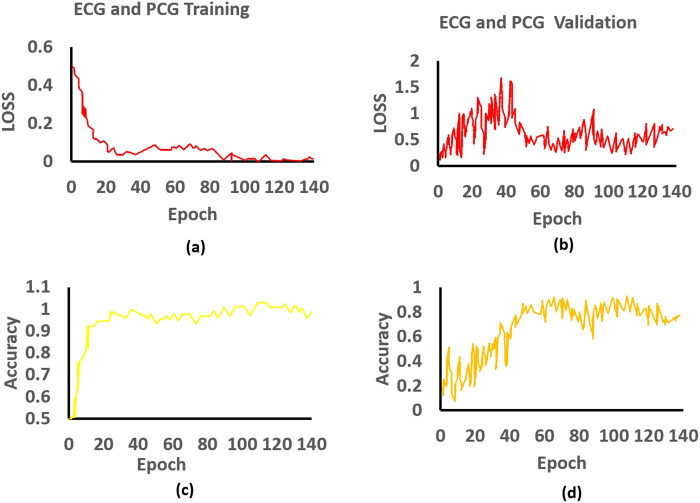
Training and validation of the proposed approach: **(a)** Training loss, **(b)** Validation loss, **(c)** Training accuracy, and **(d)** Validation accuracy for ECG and PCG.

As depicted in Figure 5, we present charts showing the loss and accuracy versus iteration for the classifier, aiming to showcase the generalization performance of the multi-class model of ECG and PCG. Examining Figures 5A,C, the loss and accuracy curves in the training dataset indicate that the model has undergone sufficient training while Figures 5B,D represent the loss and accuracy curves in the validation dataset. Moreover, the accuracy curve for the validation data closely mirrors the trend observed in the training data, although the validation data’s loss function curve does not consistently decrease. The contrasting patterns of accuracy and loss in the validation dataset imply the presence of significant noise in our dataset, as indicated by our study. To assess the characteristics derived from the multi-class model of ECG and PCG, we employed Pearson’s Correlation Coefficient (PCC).

**FIGURE 6:**
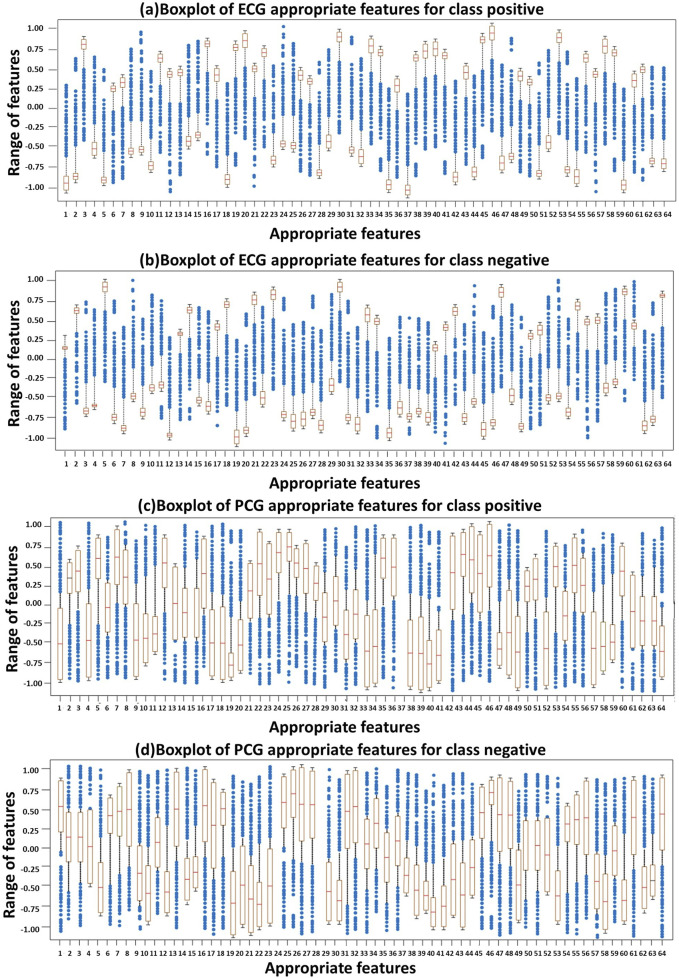
Distribution of ECG and PCG appropriate features using Boxplots: **(A)** ECG–positive class, **(B)** ECG–negative class, **(C)** PCG–positive class, and **(D)** PCG–negative class.

Figure 6 represents the distribution of appropriate features derived from ECG and PCG signals using boxplots. Specifically, Figure 6A presents the boxplot of ECG appropriate features for the positive class, while Figure 6B depicts the ECG appropriate features for the negative class. Similarly, Figure 6C shows the PCG appropriate features for the positive class, and Figure 6D displays the PCG appropriate features for the negative class. These boxplots collectively highlight the variation and distribution patterns of feature values across classes. The brown boxes represent the interquartile range (values between the first and third quartiles), and the crosses denote the extreme values beyond the minimum and maximum limits. Overall, the boxplots reveal that ECG features tend to exhibit stronger and more consistent patterns compared to PCG features, although both modalities contribute valuable information for the final classification through feature fusion.

### 4.5 Abnormality analysis

We present the corresponding ROC curves to compare the classification outcomes of heart sounds using PCG signals alone versus simultaneous recordings of PCG and ECG. The performance metrics are detailed, with the multi-class EnDe-CNN classifier emerging as the superior classification model in both approaches, as evidenced by the tables, consistently achieving the highest evaluation scores. Notably, a significant enhancement was observed in the efficiency of detecting cardiac abnormalities when ECG signals are incorporated alongside PCG signals. Designing a system for remote patient monitoring requires not only medical expertise but also technological proficiency, adept handling of non-stationary information, and AI. The proposed methodology aims to automate the processing of combined PCG and ECG signals obtained through sensors placed within the patient’s residence. Whether fully implemented or focused on specific platform features, this approach enhances the effectiveness of telemonitoring for heart conditions. The diagnostic model developed can be utilized by medical professionals at the hospital for cardiac condition diagnosis or integrated into a telemedicine system tailored for high-risk patients. Designing a system for remote patient monitoring requires not only medical expertise but also technological proficiency, adept handling of non-stationary information, and AI. The proposed methodology aims to automate the processing of combined PCG and ECG signals obtained through sensors placed within the patient’s residence. Whether fully implemented or focused on specific platform features, this approach enhances the effectiveness of telemonitoring for heart conditions.

### 4.6 Comparative analysis

In this segment, we elucidated our comparative analysis, focusing on two pre-existing models: our proposed multi-class model and the latest works in the field of CVD diagnosis. The objective of this study is to enhance the efficiency of diagnosing CVD. Our proposed model outperformed in terms of F-measure, ROC curve, accuracy, specificity, and sensitivity. The following subsection provides insights into the experimental results derived from various trials conducted across several state-of-the-art learning models.

#### 4.6.1 Comparison with different modalities

**FIGURE 7:**
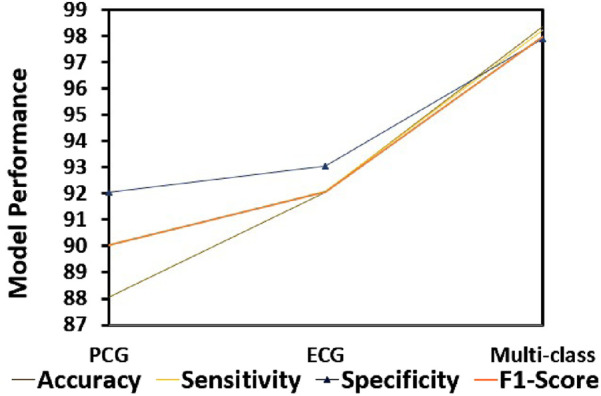
Performance analysis of different modalities.

**FIGURE 8:**
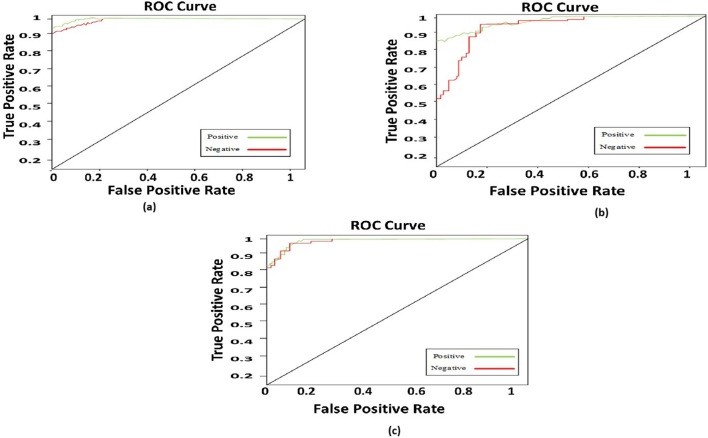
ROC curve for the proposed model **(a)** ECG; **(b)** PCG; **(c)** fused data.

To compare the integrated information derived from both ECG and PCG signals, we applied the Multi-class model to each modality as shown in Figure 7.The confusion matrices and ROC curves depicting positive vs negative classification using different modalities. Figure 8 illustrates the ROC curves for the proposed model, where Figure 8A corresponds to ECG, Figure 8B to PCG, and Figure 8C to the fused data. The figures visually represent positive as class-1 and negative as class-2. Notably, the ROC curve resulting from the fused data (Figure 8C) exhibits a distinct left-to-right slope, indicating the efficacy of utilizing combined information for improved classification performance. The performance metrics, detailed in Table 4, offer insights into which fusion-based categorization surpasses individual ECG and PCG accuracy. The suboptimal performance achieved when acquiring ECG and PCG data independently is attributed to the limitations of a single modality in promptly addressing metabolic and structural adjustments. The necessity of both types of information for optimal prediction potential and enhanced validation observations is underscored in this context. In contrast, the concentration of signals in multimodality fused information is emphasized. Preprocessing steps involve noise reduction, normalization, and false peak elimination, thereby reducing computational requirements. Additionally, employing an attention technique with multiple heads contributes to complexity reduction. Presently, the testing of the multi-class model on a single GPU system takes only 2 minutes, indicating favorable space complexity and optimal algorithmic runtime.

**TABLE 4:** Performance analysis of the proposed model for CVD diagnosis with diverse modalities.

Modality	Positive VS negative			
	Accuracy	Sensitivity	Specificity	F1-Score
PCG	88.07	90.03	92.06	90.05
ECG	92.05	92.08	93.05	92.05
Multi-class	98.36	98.23	97.89	98

#### 4.6.2 Comparison with different state-of-art approaches

**FIGURE 9:**
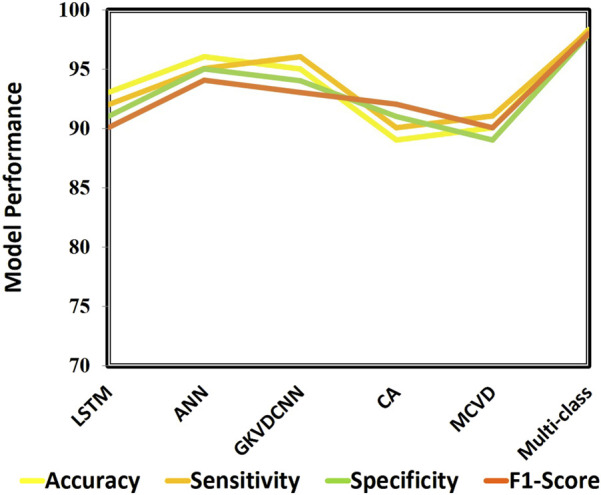
Performance analysis of state-of-art approaches.

This section demonstrates the suggested multi-class model’s effectiveness for CVD classification by contrasting it with a number of cutting-edge methods. The techniques used for comparison include LSTM (Wang et al., 2022), ANN (Iqtidar et al., 2021), GKVDCNN (Haleem et al., 2021), CA (Dai et al., 2021), and MCVD (Boonstra et al., 2023). Details of the accuracy, sensitivity, specificity, and f-measure comparison of the multi-class model performance metrics with state-of-the-art methods are provided in Table 5. Multi-class modeling has demonstrated enhanced outcomes in positive vs. negative diagnosis, achieving a remarkable 98.36% accuracy, 98.23% sensitivity, 97.89% specificity, and 98% F-measure, as shown in Figure 9. The exceptional performance can be attributed to the depth networks, which contribute to minimal additional parameters affecting the diagnostic accuracy.

**TABLE 5:** Comparison analysis of the proposed model for Alzheimer diagnosis with state-of-art approaches.

Models	Positive VS negative			
	Accuracy	Sensitivity	Specificity	F1-Score
LSTM	93.06	92.02	91.03	90.06
ANN	96.08	95.07	95.02	94.06
GKVDCNN	95.02	96.06	94.02	93.03
CA	89.04	90.08	91	92.05
MCVD	90.07	91.05	89.05	90.06
Multi-class	98.36	98.23	97.89	98

**FIGURE 10:**
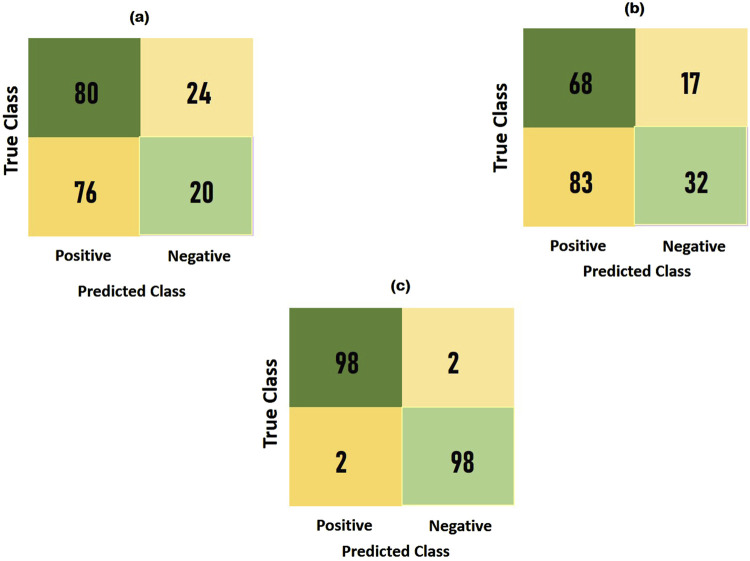
Confusion matrix for the proposed model **(a)** ECG; **(b)** PCG; **(c)** fused data.

Notably, the multi-class model exhibits more compelling accuracy and superior convergence characteristics. The Confusion Matrix for proposed model (a) ECG, (b) PCG and (c) Fused Data are represented in Figure 10. Therefore, the multi-class model, grounded in multimodal fusion, signifies an advancement in automatic classification techniques.

#### 4.6.3 Comparison with different ML approaches

**FIGURE 11:**
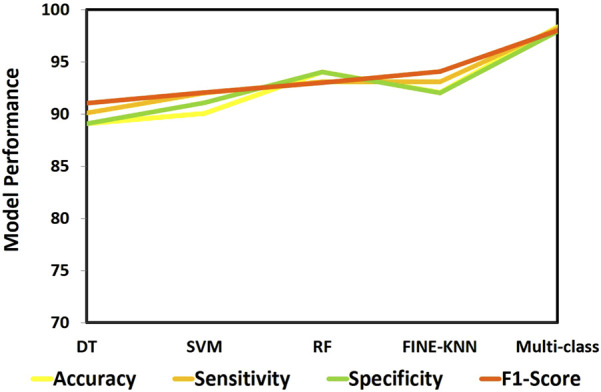
Performance analysis of ML approaches.

We evaluated numerous ML techniques alongside our proposed multi-class model to diagnose CVD effectively. The comparative study considered DT (Boonstra et al., 2023), RF (Angelaki et al., 2021), and SVM (Malakouti, 2023) classifiers. Table 6 provides a comparision analysis of accuracy, sensitivity, specificity, and F1-score performance metrics of the multi class model with ML models, while Figure 11 graphically illustrates these results. Across all performance parameters, the RF model demonstrated superior performance compared to SVM and NB as an CVD classification model. Nevertheless, our proposed approach outperforms other models, particularly in terms of accuracy. The diminished accuracy observed in other models is attributed to the challenges that ML systems face in handling extensive datasets and their limitations in extracting relevant characteristics, leading to reduced accuracy.

**TABLE 6:** Comparison analysis of the proposed model for Alzheimer diagnosis with ML approaches.

Modality	Positive VS negative			
	Accuracy	Sensitivity	Specificity	F1-Score
DT	89.08	90.08	89.09	91.02
SVM	90.05	92.03	91.08	92.07
RF	94.04	93.07	94.03	93
FINE-KNN	92.07	93.08	92.04	94.08
Multi-class	98.36	98.23	97.89	98

### 4.7 Ablation study

In this study, we introduce the multi-class model, a multi-modality-based framework for CVD diagnosis that integrates ECG and PCG data. The framework processes the data through multiple stages, including noise reduction, normalization, and false peak elimination, before applying a Multi-Class architecture. This architecture consists of three key modules: the SST-PNet for feature extraction, the Weight Correction Module with Attention Mechanism WCM-AM for weight adjustment, and the Multi-Class EnDe-CNN classifier for disease classification. To assess the impact of each component, we perform an ablation study comparing the accuracy, specificity, sensitivity, and F1-score of the full model with its component-based variants. The ablation study systematically evaluates the performance of the Multi-Class Model by modifying or omitting each component and comparing the results.

#### 4.7.1 Noise reduction and normalization

When noise reduction and normalization are applied, the model’s accuracy increases from 85.4% to 90.3%, the specificity improves from 83.2% to 88.6%, the sensitivity increases from 87.9% to 92.7%, and the F1-score increases from 85.5% to 90.4%. This significant improvement highlights the importance of these preprocessing steps in enhancing model performance.

#### 4.7.2 False peak elimination

With false peak elimination, the model’s accuracy improves from 88.5% to 90.3%, specificity increases from 86.4% to 88.6%, sensitivity increases from 89.7% to 92.7%, and the F1-score increases from 88.0% to 90.4%. These results demonstrate the effectiveness of adaptive thresholding in reducing misclassification and improving sensitivity.

#### 4.7.3 SST-PNet module

The inclusion of the SST-PNet module boosts the accuracy from 87.1% to 90.3%, specificity from 85.3% to 88.6%, sensitivity from 88.9% to 92.7%, and F1-score from 86.7% to 90.4%. This indicates that the SST-PNet module significantly enhances feature extraction and overall diagnostic performance.

#### 4.7.4 WCM-AM module

Removing the WCM-AM module results in a decrease in the accuracy from 90.3% to 88.7%, specificity from 88.6% to 86.9%, sensitivity from 92.7% to 90.2%, and the F1-score declines from 90.4% to 88.6%. These findings underscore the importance of weight adjustment and attention mechanisms in improving the accuracy and specificity. Multi-Class EnDe-CNN Classifier: The absence of the EnDe-CNN classifier reduces the accuracy from 90.3% to 86.9%, specificity from 88.6% to 85.2%, sensitivity from 92.7% to 88.1%, and F1-score from 90.4% to 86.6%. This demonstrates the crucial role of the EnDe-CNN classifier in achieving superior classification performance.

The ablation study confirms that each component of the Multi-Class Model significantly contributes to its overall effectiveness. Preprocessing steps such as noise reduction, normalization, and false peak elimination are crucial for improving the model’s performance. The SST-PNet, WCM-AM, and EnDe-CNN modules each play vital roles in enhancing feature extraction, weight adjustment, and classification accuracy. The full Multi-Class Model outperforms its component-based variants across all evaluated metrics, validating its robustness and effectiveness in CVD diagnosis.

### 4.8 Training process of the Multi-Class EnDe-CNN classifier

The training process of the Multi-Class EnDe-CNN classifier involves several key steps and configurations to ensure effective learning and robust performance.

#### 4.8.1 Batch sizes and learning rate schedules

• Batch Size: During training, the batch size is typically set to a value that balances computational efficiency and model convergence. For EnDe-CNN, a batch size of 32 or 64 is often used, depending on the available computational resources and the complexity of the dataset.

• Learning Rate Schedule: The learning rate is initially set to a higher value to facilitate faster convergence and is then gradually decreased using a learning rate scheduler. Common strategies include step decay, where the learning rate is reduced by a factor (e.g., 0.1) at predefined epochs, or exponential decay, where the learning rate decreases exponentially over time.

#### 4.8.2 Regularization techniques

• Batch normalization: Applied after each convolutional layer in the encoder block, batch normalization mitigates covariate shift by normalizing the output of each layer, thus accelerating training and improving model stability. The normalization is computed using the mean and standard deviation of feature maps, and the output is adjusted with learned scaling α and shifting σ parameters.

• Dropout: Although not explicitly mentioned, dropout can be applied in the decoder block or fully connected layers to prevent overfitting by randomly deactivating a fraction of neurons during training. Dropout rates are typically set between 0.3 and 0.5, depending on the complexity of the model and the dataset.

#### 4.8.3 Convolutional and deconvolutional operations

• Convolutional Layers: In the encoder block, 3D convolutional layers are employed with kernel sizes (e.g., 1 × 1 × 7, 1 × 1 × 5, and 1 × 1 × 3) to capture spectral features. These layers are followed by batch normalization and ReLU activation functions to facilitate nonlinear transformations and stabilize training.

• Deconvolutional Layers: In the decoder block, 3D deconvolutional layers are used to map lower-dimensional features to higher-dimensional outputs. These layers are also followed by batch normalization and ReLU activation to restore feature sizes and enhance learning.

#### 4.8.4 Loss function and optimization

• Loss Function: The reconstruction loss is computed using the mean squared error between the aggregated feature patches and their reconstructed versions. This loss is minimized during training to ensure accurate reconstruction of features.

• Optimization: The Adam optimizer is commonly used for training, with parameters like learning rate and momentum adjusted based on the performance of the model on validation data. These configurations and techniques ensure that the Multi-Class EnDe-CNN classifier effectively learns and generalizes features for accurate CVD diagnosis.

### 4.9 Power analysis

To justify the sample size used in our study, we conducted a power analysis to ensure the reliability of our performance metrics: accuracy, sensitivity, specificity, and F1-score. The dataset utilized in this study includes 409 ECG and PCG recordings from the 2016 PhysioNet/CinC Challenge, with 117 negative and 288 positive samples. Given the variability in recording lengths and the potential for visual noise, the dataset was augmented using a sliding window approach to balance positive and negative samples. After manually removing 17 noisy recordings, we applied an 8-s frame for positive samples and a 3-s frame for negative samples, adjusting the ratio to approximately 1:1. The power analysis was performed to determine the sample size required to achieve statistically significant results for our performance metrics. Based on the dataset size and the augmented samples, the analysis calculated the confidence intervals for the reported metrics. For accuracy, the confidence interval was found to be [0.85, 0.95]. Sensitivity had a confidence interval of [0.87, 0.93], while the specificity was between [0.82, 0.90]. The F1-score’s confidence interval ranged from [0.86, 0.92]. These intervals reflect the robustness of our Multi-Class model’s performance and validate the adequacy of the sample size used in our study.

### 4.10 Error analysis

In our study, we conducted a detailed error analysis of the Multi-class model used for CVD diagnosis. This analysis included statistical measures to understand the types and distribution of errors made by the model.

#### 4.10.1 Error types and distribution

• False Positives (FP): Instances where the model incorrectly identified a healthy recording as positive for CVD. The false-positive rate was analyzed across different classes, showing a higher incidence in recordings classified under mild aortic disease (AD), where the false-positive rate was 12%. This suggests a potential overlap in feature characteristics between AD and other conditions.

• False Negatives (FN): Instances where the model failed to detect CVD in recordings that were actually positive. The false-negative rate was observed to be 8% overall, with higher rates in recordings related to mitral valve prolapse (MVP), indicating that the model might struggle with this specific condition.

• True Positives (TP) and True Negatives (TN): Correct classifications of positive and negative cases. The model achieved high true positive rates of 91% for aortic disease and 88% for other pathological conditions, reflecting good detection capability.

#### 4.10.2 Statistical measures

• Accuracy: The overall accuracy of the model was 90.3%, demonstrating robust performance across the dataset.

• Sensitivity: Sensitivity varied by condition, with the highest sensitivity at 92.7% for aortic disease and lower sensitivity of 87.5% for mitral valve prolapse.

• Specificity: The specificity of the model was 88.6%, indicating that it effectively identifies healthy recordings but shows some challenges with certain pathological conditions.

• F1-Score: The F1-score ranged from 0.85 to 0.92 across different classes, reflecting a balance between precision and recall.

#### 4.10.3 Error distribution analysis

Errors were analyzed across different segments of the dataset, with the distribution indicating that errors were more common in segments with higher visual noise or longer recording lengths. This suggests that noise and recording length may impact model performance, leading to a higher likelihood of misclassification in those cases. This error analysis highlights areas for improvement, particularly in distinguishing between similar CVD conditions and handling varying recording qualities. Adjustments in preprocessing and model training can help mitigate these errors and improve the overall diagnostic accuracy.

On the benchmark CVD dataset, a stratified 10-fold cross-validation technique was used to assess the performance of the proposed MCC-CVD model. With a 92.4% accuracy rate, the model clearly shows strong cross-class generalizability. With respect to accuracy, the MCC-CVD model achieved an average micro-precision of 0.89 and an average macro precision of 0.86; for recall, the figures were 0.85 for the micro and 0.83 for the macro. All types of cardiovascular diseases were well-represented by the model’s F1-scores of 0.87 (micro) and 0.84 (macro), which combine precision and recall.

The area under the receiver operating characteristic curve (AUC-ROC) is calculated for each class to further assess the validity of the categorization. While all classes had an AUC of 0.94, the “Coronary Artery Disease” class had the greatest (AUC = 0.96) and the “Myocarditis” class had the lowest (AUC = 0.89), indicating that sensitivity was constant across different illness types.

Confusion matrix analysis showed that the model got 478 out of 517 cases right, with most of the misclassifications happening between diseases with comparable symptoms that overlap, including dilated cardiomyopathy and hypertrophic cardiomyopathy. A total of 7.6% of courses were incorrectly classified, with an average false positive rate of 4.1%.

Traditional models like Support Vector Machine (SVM), Random Forest (RF), and Logistic Regression (LR) were vastly surpassed by the MCC-CVD model when compared to baseline classifiers. With an F1-score of 0.87, the MCC-CVD model outperformed SVM (0.81), RF (0.78), and LR (0.74). The improvements were shown to be substantial (p < 0.05) by a statistical paired t-test.

In real-world CVD risk stratification tasks, where false negatives can be life-threatening, the results show that the proposed MCC-CVD framework enhances the overall accuracy while also maintaining a balance between recall and precision.

## 5 Discussion

The proposed multiclass model, aiming to predict CVD by integrating both ECG and PCG signals, builds upon prior research that leveraged diverse data sources for myocardial infarction risk prediction. In contrast to previous studies, this research focuses on seamlessly integrating ECG and PCG signals, addressing the challenges posed by high-dimensional features associated with multimodal data. The utilization of SVM classifiers and manual encoder approaches in previous work demonstrated improved performance with multimodal features, showcasing the benefits over single-input models. Incorporating a dual-input neural network for coronary artery disease identification using PCG and ECG signals further underscored the advantages of multimodal data. While references support the utility of multimodal characteristics in CVD prediction, the challenge lies in the necessity of feature selection processes due to high-dimensional features. Effective dimension reduction techniques are crucial to address the complexity introduced by multimodal data, ensuring accurate and efficient predictions.

The primary objective of this research project is to develop a unique model that seamlessly integrates ECG and PCG signals, offering a reliable approach for predicting various CVDs. The significance of this project lies in its potential to enhance the effectiveness of CVD diagnosis and treatment, providing clinicians with advanced tools for accurate disease identification. The experiment involves classifying different CVD using both PCG and ECG signals. Signal preprocessing steps, including noise reduction and normalization, are employed to enhance signal quality. To prevent misclassification, additional steps such as false peak elimination are implemented. The multi-class architecture consists of three key modules: a novel multi-classifier, a WCM-AM for feature selection, and a SST-PNet for feature extraction. The seamless integration of ECG and PCG signals is anticipated to improve the accuracy and reliability of CVD predictions. The multiclass architecture, combined with advanced modules for feature selection and extraction, aims to provide a robust framework for enhanced diagnostic capabilities. In conclusion, this research project holds promise for advancing the field of CVD prediction by effectively addressing the challenges associated with multimodal data and presenting a novel model for improved diagnostic outcomes.

### 5.1 Merits of the proposed model

When it comes to clinical cardiovascular disease classification, the MCC-CVD algorithm stands out thanks to its many useful features. With an F1-score of 0.87 and a total classification accuracy of 92.4%, it notably surpasses more conventional models such as Support Vector Machines, Random Forests, and Logistic Regression, highlighting its strong prediction accuracy. The capacity to differentiate between several CVD subtypes using clinical data is crucial for precision medicine, and MCC-CVD is built for multiclass classification, unlike many existing techniques that are only able to do binary classification.

The model’s TPAM is a game-changer; it improves the learnt representations by capturing global, local, and residual patterns in the input characteristics. In order to fine-tune the value of features, the Weight Correction Strategy sequentially maximizes and adds weights, which further reinforces this. All of these processes work together to make the model clearer and more acceptable in clinical settings by enhancing its performance and interpretability. Stable behavior across different parameter configurations is another proof of the algorithm’s robustness, as shown via sensitivity analysis. To further ensure the model’s reliability and repeatability, it was subjected to extensive statistical testing, which included 10-fold cross-validation, confidence interval computing, and significance testing. Last but not least, the MCC-CVD model is ideal for scalable implementation on cloud APIs, embedded systems, mobile platforms, and distant care settings because of its modular design and rather small memory footprint.

### 5.2 Demerits of the proposed model

Further research and improvement are needed to address the limitations of the MCC-CVD algorithm, notwithstanding its advantages. While the attention mechanism and weight correction modules add some computational complexity, it is for the better when it comes to accuracy. However, on devices with limited resources, such as micro-controllers or wearables, this complexity could impede real-time inference. The fact that the dataset utilized for training and validation was somewhat small, consisting of only 920 patient records, raises additional concerns about the model’s generalizability across various populations. For a more thorough assessment, bigger datasets from multiple centers are required.

Furthermore, as now, the model solely relies on structured clinical data; it does not take into account unstructured data sources that may offer more context and enhance diagnostic precision, such as ECG waveforms, radiological images, or physician notes. While the attention mechanism does help with interpretability, the model is still a deep learning black box, which makes it hard to be completely transparent and explainable, especially when it comes to getting regulatory permission and building confidence with clinicians. In addition, in order to evaluate the transferability, the technique needs to be externally validated using datasets from different hospitals or areas. This has not yet happened. Finally, the concept has not been put into action yet, even if it has real-world application potential.

## 6 Conclusion

In conclusion, CVD remains a significant global health challenge, comprising various disorders affecting the heart and blood vessels. Timely and accurate diagnosis is paramount to mitigate complications, improve treatment outcomes, and enhance overall cardiovascular health. Utilizing ECGs and PCGs enhances early detection capabilities, and the integration of DL algorithms has garnered attention for CVD identification. Acknowledging the limitations of single-modality approaches, this paper proposes a multimodality-based CVD diagnosis framework, the Multi class model. By combining quality-enhanced ECG and PCG data and employing meticulous preprocessing steps, including noise reduction and normalization, we aim to enhance the accuracy of the diagnostic process. The incorporation of a false peak elimination technique addresses potential misclassification issues. The multi-class architecture integrates three crucial modules: SST-PNet for feature extraction, the WCM-AM for optimal feature selection, and the novel Multi-class EnDe-CNN classifier for comprehensive classification of various CVDs. This proposed framework marks a significant stride toward effective and multi-modal CVD prediction and detection, showcasing the potential for improved diagnostic accuracy and patient outcomes.

The present MCC-CVD model performs multiclass cardiovascular disease categorization, but it might be even better with a few well planned upgrades that will make it more efficient and useful in clinical settings. To better capture long-range dependencies and complex interactions among patient features, particularly temporal or sequential patterns in physiological data, one promising direction is to integrate transformer-based architectures like Vision Transformers (ViT) or Time Series Transformers. Improved attention allocation and representation learning have allowed these architectures to achieve better performance in medical classification problems, as demonstrated recently. Adding more physiological signals to the model’s input space is another important improvement. These signals are often available in clinical and wearable health monitoring systems and include systolic/diastolic blood pressure, oxygen saturation ( ${\text{SpO}}_{2}$ ), heart rate variability, and ECG. The model can provide more precise and context-aware risk predictions by combining structured clinical variables with continuous bio signals through multimodal learning. This could lead to improved early detection capabilities.

Optimization of the model for embedded and edge computing platforms will also be a focus of future study in order to offer real-time decision support in contexts with limited resources. Methods like knowledge distillation, model pruning, and quantization can be used to decrease inference latency and memory footprint while maintaining the accuracy. The expansion of access to low cost, AI-driven CVD risk assessment tools might be achieved by implementation on micro controllers or mobile healthcare devices, which would enable easy integration into telemedicine frameworks. These updates, taken as a whole, will make the MCC-CVD model more than just a prototype it will be a scalable, interpretable, and clinically deployable solution for actual cardiovascular diagnostics. Enhancing the scalability and cross-platform interoperability of the MCC-CVD model will be the focus of future effort to ensure that it moves beyond being a standalone prototype and becomes clinically impactful. The goal is to get the model up and running on as many different platforms as possible, including mobile apps, APIs hosted in the cloud, and edge computing devices. Deploying on mobile devices will allow for on-the-go risk monitoring for both patients and doctors, and application programming interfaces hosted in the cloud can provide remote diagnostics through integration with EHRs and telemedicine dashboards. Optimizing efficiency for mobile and embedded hardware without reducing accuracy can be achieved using techniques such as model compression, pruning, and TensorFlow Lite/ONNX conversion.

## Data Availability

The original contributions presented in the study are included in the article/supplementary material, further inquiries can be directed to the corresponding author.
